# Magnetoelectric Transverse Gradient Sensor with High Detection Sensitivity and Low Gradient Noise

**DOI:** 10.3390/s17112446

**Published:** 2017-10-25

**Authors:** Mingji Zhang, Siu Wing Or

**Affiliations:** 1 Department of Electrical Engineering, The Hong Kong Polytechnic University, Hung Hom, Kowloon, Hong Kong, China; mingji.zhang@connect.polyu.hk; 2 Hong Kong Branch of National Rail Transit Electrification and Automation Engineering Technology Research Center, Hong Kong, China

**Keywords:** ambient noise suppression, baseline, magnetic field gradient, magnetoelectric effect, transverse gradient sensor

## Abstract

We report, theoretically and experimentally, the realization of a high detection performance in a novel magnetoelectric (ME) transverse gradient sensor based on the large ME effect and the magnetic field gradient (MFG) technique in a pair of magnetically-biased, electrically-shielded, and mechanically-enclosed ME composites having a transverse orientation and an axial separation. The output voltage of the gradient sensor is directly obtained from the transverse MFG-induced difference in ME voltage between the two ME composites and is calibrated against transverse MFGs to give a high detection sensitivity of 0.4–30.6 V/(T/m), a strong common-mode magnetic field noise rejection rate of <−14.5 dB, a small input-output nonlinearity of <10 ppm, and a low gradient noise of 0.16–620 nT/m/$\sqrt{\mathrm{Hz}}$ in a broad frequency range of 1 Hz–170 kHz under a small baseline of 35 mm. An analysis of experimental gradient noise spectra obtained in a magnetically-unshielded laboratory environment reveals the domination of the pink (1/*f*) noise, dielectric loss noise, and power-frequency noise below 3 kHz, in addition to the circuit noise above 3 kHz, in the gradient sensor. The high detection performance, together with the added merit of passive and direct ME conversion by the large ME effect in the ME composites, makes the gradient sensor suitable for the passive, direct, and broadband detection of transverse MFGs.

## 1. Introduction

Magnetic field gradients (MFGs) are the variations of magnetic fields per unit length in three-dimensional space [1]. Magnetic gradient sensors are a key member of the magnetic sensor family, featuring a unique and strong ability to suppress ambient (i.e., common-mode) noise by detecting MFGs [1,2]. They generally consist of two magnetic field sensors spatially separated by a baseline to detect MFGs by differencing the output signals of the two magnetic field sensors over the baseline. This distinct noise-suppression ability has enabled magnetic gradient sensors to play an increasingly crucial role in outdoor environments and critical applications involving high ambient noise. Some typical examples include geomagnetic mapping, magnetic anomaly detection, magnetic navigation, magnetic object positioning, electric grid current sensing, transportation condition monitoring, medical diagnosis, and non-destructive evaluation [1,2,3,4,5,6]. To this extent, it is highly desirable to develop passive (i.e., power-free), small-scale, and high-performance magnetic gradient sensors possessing simultaneously high detection sensitivity, strong common-mode magnetic field noise rejection rate, small input-output nonlinearity, and low gradient noise over a broad frequency range under a small baseline.

To date, most of the existing magnetic gradient sensors use fluxgate elements, Hall elements, and superconducting quantum interference devices (SQUIDs) as the magnetic field sensors [7,8,9,10,11,12]. The fluxgate gradient sensors can generally achieve a high detection sensitivity of ~10 V/(T/m) and a low gradient noise of ~10 nT/m/$\sqrt{\mathrm{Hz}}$ (at 1 Hz) at the expense of requiring an AC excitation current supply, a low-noise low-drift signal modulator, and a large baseline (>0.1 m) to sustain their operations [7,8]. The Hall gradient sensors, because of the inherently weak Hall voltages (<10 V/T) and high semiconductor noise (>0.1 μV/$\sqrt{\mathrm{Hz}}$) in their Hall elements, usually have a lower detection sensitivity of <0.1 V/(T/m) and a higher gradient noise of >1 μT/m/$\sqrt{\mathrm{Hz}}$ (at 1 Hz). Moreover, they need a highly stable DC current supply to excite the Hall effect and a high-quality signal conditioner to process the weak Hall voltages under noise [9,10,11]. The SQUID gradient sensors are sensitive enough to detect MFGs down to ~10 fT/m with a very low gradient noise of ~10 fT/m/$\sqrt{\mathrm{Hz}}$ (at 1 Hz). However, the practical necessity of equipping a bulky cryogenic system to assure superconductivity significantly restricts them to be laboratory equipment [12,13].

ME composites, owing to their unique ability to directly induce interestingly large ME voltages in excess of 100 times over the Hall voltages without the support of external power supplies, signal conditioners, and/or other auxiliary means as normally required in the fluxgate elements, Hall elements, and SQUIDs, have received considerable research and application attention as highly-sensitive passive sensors for AC magnetic field and electric current detections over the past decade [14,15,16,17,18,19]. Recently, reports connecting between ME composites and MFG detection have occurred [20,21,22,23,24]. MFG-based DC ME sensors have been studied by exciting Terfenol-D/PZT or Ni/PZT ME heterostructures with an AC resonance voltage to induce an AC magnetic field in accordance with the converse ME effect and by interacting this AC magnetic field with the DC magnetic field to be measured so as to produce an MFG and, hence, a shift in resonance frequency and a change in both output voltage and phase angle [20,21,22,23,24]. An ME gradient technique for suppressing ambient noise has been investigated by using two Metglas/PZT/Metglas trilayer ME composites to detect magnetic fields at two different locations on the basis of the ME effect and by feeding the corresponding ME voltages into two separate channels of a data logger interfaced with a MATLAB-based differential program for post-processing [25]. While effective, there does not appear to be any solid work on applying or advancing the ME gradient technique to realize standalone (i.e., intrinsic) ME gradient sensors for the passive and direct detection of MFGs into electrical voltages in the absence of any auxiliary software and/or hardware for post-processing differentiation.

In view of the above, we have developed and reported the basic detection performance of a small-scale and standalone ME transverse gradient sensor capable of detecting transverse MFGs into electrical voltages by combining the passive and direct ME conversion ability enabled by the ME effect with the ambient noise suppression ability facilitated by the MFG technique in a pair of plate-shaped and magnet-biased Terfenol-D/PZT/Terfenol-D trilayer ME composites [26]. This gradient sensor is essentially a transverse-type MFG sensor for detecting MFGs transverse to its length in the axial direction, which is analogous to transverse-type Hall probes for detecting magnetic fields, transverse to their length in the axial direction. In fact, the development of the transverse gradient sensors is important to compromise the application inconvenience and limitation in axial gradient sensors, such as detection of MFGs in gaps with limited space. It can also open up opportunities to furnish full tensor gradient sensors by combining transverse gradient sensors with axial gradient sensors.

In this paper, the theoretical and experimental details on the design, modeling, and evaluation of the ME transverse gradient sensor are disclosed. The governing equations, boundary conditions, and modeling process for implementing multiphysics finite element analysis (FEA) are provided. A representative gradient sensor having a baseline as small as 35 mm is demonstrated, and its performance is characterized in a magnetically-unshielded laboratory environment, in terms of detection sensitivity, common-mode magnetic field noise rejection rate, input-output nonlinearity, and gradient noise. A comprehensive analysis of the experimental gradient noise spectra is carried out to study the contribution of various noise types to the ambient noise associated with the gradient sensor. 

## 2. Configuration, Structure, and Prototype

**Figure 1:**
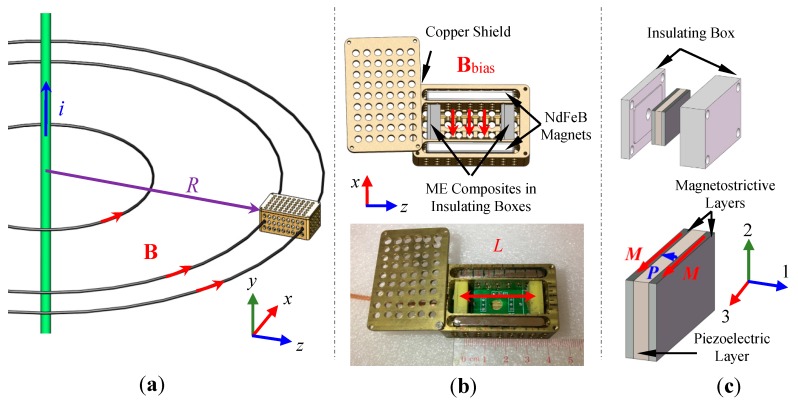
Proposed ME transverse gradient sensor: (**a**) configuration under MFGs of a current-carrying cable; (**b**) structure and prototype; and (**c**) an ME composite and its insulating box, where *M* denotes the magnetization direction of the MS layers and *P* indicates the polarization direction of the PE layer.

Figure 1 shows the configuration, structure, and prototype of the proposed ME transverse gradient sensor. The gradient sensor consists of a pair of plate-shaped Terfenol-D/PZT/Terfenol-D trilayer ME composites mechanically enclosed in two separate insulating boxes with Superwool surrounding and magnetically biased by a pair of NdFeB magnets in a copper shield. The two mechanically enclosed ME composites were oriented in the transverse (*x*-) direction, separated with a baseline (*L* = 35 mm) in the axial (*z*-) direction, and biased by the two NdFeB magnets with an average DC magnetic field ($\left|{\bar{B}}_{\mathrm{bias}}\right|$ = 56 mT) in the transverse (*x*-) direction, all with reference to the length of the gradient sensor in the axial (*z*-) direction. The copper shield was grounded and drilled with holes of 3.2 mm diameter in an array form to minimize the influence of ambient electric fields. The ME composites were prepared by bonding a layer of PZT (Pb(Zr, Ti)O_3_, Ceram-Tec P8) piezoelectric (PE) ceramic plate of 12 mm length, 6 mm width, and 1 mm thickness between two layers of [112]-textured Terfenol-D (Tb_0.3_Dy_0.7_Fe_1.92_, Baotou Rare Earth, Baotou, China) magnetostrictive (MS) alloy plates of the same dimensions in the thickness (1-) direction (Figure 1c). The magnetization (*M*) direction of the Terfenol-D plates and the polarization (*P*) direction of the PZT plate were oriented in their length (3-) and thickness (1-) directions, respectively. A total of ten ME composite samples were prepared, and two of the most similar ones in terms of ME properties were deliberately selected to form the ME composite pair in the gradient sensor so as to reduce the difference in ME voltage coefficient to <3%. Two NdFeB magnet plates, each of 40 mm length, 15 mm width, and 5 mm thickness, were arranged in the axial (*z*-) direction to bias the ME composites along their length (3-) direction at $\left|{\bar{B}}_{\mathrm{bias}}\right|$ = 56 mT. The negative electrode surface of the PZT plate in the two ME composites was connected together to form a back-to-back capacitor configuration, while the positive electrode surface of the first and second PZT plates was connected to the signal core and ground shield of the coaxial cable with BNC termination, respectively. Therefore, the output voltage of the gradient sensor as measured at the BNC termination was essentially the transverse MFG-induced difference in ME voltage between the two ME composites and was directly calibrated against transverse MFGs to give the detection sensitivity characterized by a unit voltage output per unit transverse MFG input. It is noted that our gradient sensor is essentially a small-scale and standalone device, which completely gets rid of the software and hardware necessarily for post-processing differentiation in the previously reported ME gradient technique [25].

## 3. Working Principle

The working principle of the ME transverse gradient sensor in Figure 1 can be described by a transverse MFG-induced difference in ME voltage between the two ME composites on the basis of the ME effect and the MFG technique. Accordingly, the dynamic coupling between the MS and PE effects in the MS (i.e., Terfenol-D) and PE (i.e., PZT) plates in the two ME composites, as well as the direct difference in ME voltage between the ME composites are physically important.

### 3.1. Governing Equations of Magnetic Fields

The magnetic fields (**B**) in the FEA domains are generally governed by Maxwell’s equations in a frequency-dependent manner as follows:

(1a) B=∇×A,

(1b) $$\nabla \times {\left(\mu_{0}\mu_{r}\right)}^{-1}B=J,$$

(1c) $$E_{\;}=-j\left(2\pi f\right)A,$$

(1d) $$J=\sigma E_{\;}+j\left(2\pi f\right)D.$$

where **B** is the curl of the magnetic vector potential (A) in Equation (1a) and is related to the current density (J), the vacuum permeability ($\mu_{0}$), and the relative permeability ($\mu_{r}$) by Ampere’s Law in Equation (1b). A in Equation (1a) is related to the electric field (E) by Faraday’s Law in Equation (1c). J in Equation (1b) is formed by the conductive current density (σE) and the displacement current density (j(2πf)D) in Equation (1d), where σ is the electrical conductivity and f the frequency.

In the FEA of current-carrying cables, **B** in Equation (1b) can be obtained by implementing current density boundary condition to quantify J. For current-carrying coils, **B** can be acquired by expressing J as:

(2) $$J=\frac{NI_{\mathrm{coil}}}{A}e_{\mathrm{coil}}$$

where $I_{\mathrm{coil}}$ is the current amplitude per unit turn, N the total number of turns, A the cross-section area, and $e_{\mathrm{coil}}$ the geometric shape vector. 

### 3.2. Elastic Motions in ME Composites

Since the two ME composites are mechanically enclosed in two separate insulating boxes with Superwool surrounding for thermal insulation, the MS static strain of the MS plates due to **B**_bias_ can be considered as the main contributor to the total static strain ($\varepsilon_{0}$) in the ME composites. By defining the MS dynamic strain ($\varepsilon_{\mathrm{MS}}$) as $\varepsilon_{\mathrm{MS}}=1/2\left[{\left(𝛻u_{\mathrm{MS}}\right)}^{\prime }+𝛻u_{\mathrm{MS}}\right]$, the *f* dependence of the dynamic mechanical displacement of the MS plates ($u_{\mathrm{MS}}$) in the ME composites can be expressed as:

(3) $$-\rho {\left(2\pi f\right)}^{2}u_{\mathrm{MS}}=𝛻\cdot \left[C^{B}:\left(\varepsilon_{\mathrm{MS}}-\varepsilon_{0}\right)\right],$$

where ρ is the density, $C^{B}$ the fourth-order elastic matrix of the MS plates under a constant static magnetic field, and the symbol ‘**:**’ represents the double-dot product operator. 

As the ambient electric fields are minimized by the copper shield, their effects on the PE static and dynamic strains of the PE plate can be neglected. By defining the PE dynamic strain ($\varepsilon_{\mathrm{PE}}$) as $\varepsilon_{\mathrm{PE}}=1/2\left[{\left(𝛻u_{\mathrm{PE}}\right)}^{\prime }+𝛻u_{\mathrm{PE}}\right]$, the *f* dependence of the dynamic mechanical displacement of the PE plate ($u_{\mathrm{PE}}$) in the ME composites can be written as:

(4) $$-\rho {\left(2\pi f\right)}^{2}u_{\mathrm{PE}}=𝛻\cdot \left[C^{E}:\left(\varepsilon_{\mathrm{PE}}-\varepsilon_{0}\right)\right]$$

where $C^{E}$ is the fouth-order elastic matrix of the PE plate under constant static electric field.

### 3.3. Nonlinear Constitutive Relations of MS Plates

To enable FEA on the MS plates, the nonlinear constitutive relations between the total magnetization ($M_{t}$) and the total magnetic field ($B_{t}$) can be established from saturation magnetization ($M_{s}$) and a hyperbolic tangent function of magnetic susceptibility ($\chi_{m}$), $\mu_{0}$, $\mu_{r}$, and $M_{s}$ to be:

(5) $$M_{t}=M_{s}\tanh\left(\frac{\chi_{m}}{\mu_{0}\mu_{r}}\cdot \frac{B_{t}}{M_{s}}\right)\left(\frac{B_{t}}{\left|B_{t}\right|}\right)$$

where $B_{t}$ is the sum of B and $B_{\mathrm{bias}}$, and $M_{t}$ the sum of B-induced AC magnetization (**M**) and $B_{\mathrm{bias}}$-induced DC magnetization ($M_{\mathrm{dc}}$) [27].

Due to the anisotropic nature of the MS plates, $\varepsilon_{\mathrm{MS}}$ in Equation (3) can be formulated as:

(6) $$\varepsilon_{\mathrm{MS}}=\frac{3}{2}\frac{1}{M_{s}^{2}}\left[\lambda_{001}\mathrm{dev}\left(m_{p}m_{q}\right)+\left(\lambda_{111}-\lambda_{001}\right)\underset{p\neq q}{\sum }m_{p}m_{q}e_{p}e_{q}\right]$$

where $m_{p}$ and $m_{q}$ are the unit directional vectors of $M_{t}/M_{s}$ in the Cartesian coordinate system (*p*, *q* = 1, 2, 3) in Figure 1c; $e_{p}$ and $e_{q}$ are the directional cosines between the 1-, 2-, 3- and *x*-, *y*-, *z*- directions in Figure 1; and the symbol ‘dev’ denotes the deviatoric projection operator [28].

### 3.4. Constitutive Relations of PE Plate

Due to the mechanical bonding between the MS and PE plates in the ME composites, $\varepsilon_{\mathrm{MS}}$ in Equations (3) and (6) leads to $\varepsilon_{\mathrm{PE}}$ in Equation (4) which, in accordance with the PE constitutive relations, induces an electric displacement field (D) in the PE plate as:

(7) $$D=e^{E}\varepsilon_{\mathrm{PE}}+\epsilon_{0}\epsilon_{r}E+P$$

where $\epsilon_{0}$ is the vacuum permittivity, $\epsilon_{r}$ is the relative permittivity matrix, **P** is the remnant polarization vector, and $e^{E}$ is the PE coupling matrix in stress–charge form under a constant electric field [29]. By constraining D in Equation (7) with the following charge conservation law:

(8) ∇·D=0

and using the boundary conditions to be described in Section 3.5, the open-circuit electric potential distribution (V) of the PE plate can be obtained by:

(9)  E=−∇V

### 3.5. Boundary Conditions

To allow FEA on **B**, the outermost boundaries of the whole FEA domains can be taken by magnetic insulation constraint as:

(10) n×A=0

where n is the normal vector of a boundary. Equation (10) is a Dirichlet-type boundary condition for improving convergence in calculation.

The Superwool surrounding for the ME composites can be approximated by applying free boundary condition to the outermost boundaries of the ME composites. The mechanical bonding between the MS and PE plates can be described using continuous boundary condition at the adjacent boundaries between the MS and PE plates as:

(11) $$u_{\mathrm{PE}}=u_{\mathrm{MS}}$$

The two ME composites with a back-to-back capacitor configuration can be modeled by applying floating boundary condition in Equation (12), ground boundary condition in Equation (13), and terminal boundary condition in Equation (14) to the negative electrode surfaces of the two PE plates, the positive electrode surface of one PE plate, and the positive electrode surface of another PE plate, respectively:

(12) $$\overset{\;}{\underset{\Gamma }{\int }}D\cdot ndS=0$$

(13) V=0

(14) $$\overset{\;}{\underset{\Gamma }{\int }}D\cdot ndS=Q$$

where the symbol ‘Γ’ indicates the surface geometry of a positive electrode, dS is the surface element of a positive electrode, and *Q* the total charge accumulation on each positive electrode surface. The output voltage of a gradient sensor ($V_{G}$) can be expressed as:

(15) $$V_{G}=\omega R_{L}Q$$

where $R_{L}$ ≥ 1 MΩ is the input resistance of external circuit under open-circuit condition.

The ME voltages of the two ME composites ($V_{M,A}$ and $V_{M,B}$) can be obtained by surface average probe function. Their difference leads to:

(16) $$V_{G}=V_{M,A}-V_{M,B}$$

It is noted that $V_{G}$ obtained from Equation (16) is numerically the same as that acquired from Equation (15). 

The ME voltage coefficients of the two ME composites ($\alpha_{i,A}\;\mathrm{and}\;\alpha_{i,B}$, V/T) oriented in the *i*-direction (i=x, y, z) can be characterized by a unit ME voltage output per unit magnetic field input as follows:

(17a) $$\alpha_{i,A}=\frac{dV_{M,A}}{dB_{i,A}}$$

(17b) $$\alpha_{i,B}=\frac{dV_{M,B}}{dB_{i,B}}$$

The tensorial form of MFGs (G) is generally expressed by the vector differential of B over spatial vector (r) as:

(18) $$G=\frac{\partial B}{\partial r}$$

The components of G ($G_{ij}$) can be obtained by spatially differencing $B_{i,A}$ and $B_{i,B}$ over the baseline ($L_{j}$), giving:

(19) $$G_{ij}=\frac{B_{i,A}-B_{i,B}}{L_{j}}=\frac{1}{\alpha_{i}}\frac{V_{M,A}-V_{M,B}}{L_{j}}\;\left(i,j=x,y,z\right)$$

Equation (19) provides a theoretical distinction between the axial (i=j) and transverse (i≠j) gradient sensors. From Equations (15) and (19), the detection sensitivity of a gradient sensor ($S_{G,ij}$), which is characterized by a unit voltage output per unit MFG input, can be written as:

(20) $$S_{G,ij}=\frac{dV_{G}}{dG_{ij}}=\alpha_{i}L_{j}$$

### 3.6. Ambient Noise Suppression

In ME transverse gradient sensors, $\alpha_{i,A}\;\mathrm{and}\;\alpha_{i,B}$ in Equations (17) can be combined with $V_{G}$ in Equation (16) to give the transverse MFG-induced difference in ME voltage between the two ME composites as:

(21) $$V_{G}=\alpha_{i,A}B_{i,A}-\alpha_{i,B}B_{i,B}$$

By taking into account the effects of voltage noises ($v_{A}\sqrt{∆f}$ and $v_{B}\sqrt{∆f}$) on the ME voltages ($\alpha_{i,A}B_{i,A}$ and $\alpha_{i,B}B_{i,B}$), $V_{G}$ in Equation (21) can be modified as:

(22) $$V_{G}=\left(\alpha_{i,A}B_{i,A}+v_{A}{\sqrt{∆f}}^{\;}\right)-\left(\alpha_{i,B}B_{i,B}+v_{B}{\sqrt{∆f}}^{\;}\right)$$

where $v_{A}$ and $v_{B}$ are the voltage noise densities due to various types of ambient noise, including magnetic field noise, electric field noise, vibration noise, pink (1/*f*) noise, dielectric loss noise, and power-frequency noise [30]. 

In our design, the difference in ME voltage coefficient of the two ME composites is kept to <3% (Section 2) so that $\alpha_{i,A}≅\alpha_{i,B}$ holds true in Equation (22) and the ambient noise has almost the same level of influence on each ME composite ($v_{A}\sqrt{∆f}≅v_{B}\sqrt{∆f}$). Thus, $V_{G}$ in Equation (22) can be further modified by combining Equation (16) with Equation (19) to form:

(23) $$V_{G,ij}≅\alpha_{i}L_{j}G_{ij}.$$

In practice, the relation of $v_{A}\sqrt{∆f}≅v_{B}\sqrt{∆f}$ will be weakened at high frequencies because of the phase-lag between $v_{A}\sqrt{∆f}$ and $v_{B}\sqrt{∆f}$. This is especially obvious for the pink (1/*f*) noise density ($v_{1/f}$) and the dielectric loss noise density ($v_{\mathrm{loss}}$) as follows [30,31]:

(24) $$v_{1/f}=\frac{1}{\omega C}\sqrt{\frac{4kT∆f}{R_{L}}}$$

(25) $$v_{\mathrm{loss}}=\sqrt{\frac{4kT\tan\delta ∆f}{\omega C}}$$

where δ, k, and T are the dielectric loss factor, the Boltzmann’s constant, and the temperature, respectively. 

In the reported ME gradient technique for ambient noise suppression, two ME composites were used to produce two ME voltages in response to magnetic fields at two different locations [25]. The two field noises ($h_{A}$ and $h_{B}$, T/$\sqrt{\mathrm{Hz}}$) can be obtained by, respectively, measuring the voltage noise densities of the two ME composites ($v_{A}$ and $v_{B}$, V/$\sqrt{\mathrm{Hz}}$) over $\alpha_{i}$ using:

(26) $$h_{A}=v_{A}/\alpha_{i,A}$$

(27) $$h_{B}=v_{B}/\alpha_{i,B}$$

For our ME transverse gradient sensor, the gradient noise ($g_{\;}$, T/m/$\sqrt{\mathrm{Hz}}$) can be evaluated by directly measuring the voltage noise density of the gradient sensor ($v_{G}$, V/$\sqrt{\mathrm{Hz}}$) over $S_{G,xz}$ using:

(28) $$g=v_{G}/S_{G,\mathrm{xz}}$$

## 4. Performance Evaluation, Results, and Discussion

In this section, the directional subscripts *i*, *j* used in Section 3 are designated to be *x* and *z* in regard to the structure and configuration of our ME transverse gradient sensor in Figure 1a. For simplicity, we use G, L, $\alpha_{A\;}$,$\;\alpha_{B\;}$, $S_{G}$, and $V_{G}$ to represent $G_{xz}$, $L_{z}$, $\alpha_{x,A}$, $\alpha_{x,B}$, $S_{G,xz}$, and $V_{G,xz}$ in the following discussion. In Section 4.1 and Section 4.2, the FEA is implemented in a commercial multiphysics simulation software. (COMSOL Multiphysics^®^ version 5.2a released by COMSOL Inc. Stockholm, Sweden) The FEM models are implemented in five steps, including: (1) building geometry in accordance with experimental configuration; (2) mesh generation and optimization; (3) setting material properties; (4) multiphysics coupling of magnetic field, solid mechanics, electrostatics, and electrical circuit modules; and (5) configuring solvers and processing calculated results. The governing equations of the above modules, which have been presented and discussed in Section 3, are applied to create two FEA models for the evaluation of the ME voltage coefficients of the two ME composites and for the calibration of the detection sensitivity of the gradient sensor in accordance with the respective experimental arrangements using the frequency-dependent solver of COMSOL Multiphysics^®^. The material parameters of Terfenol-D and PZT, as shown in Table 1 and Table 2, respectively, are employed for the FEA. The calculated results are compared with the measured results for further discussion. In Section 4.3, the voltage noise density spectra and gradient noise spectra of the two ME composites and the gradient sensor are evaluated experimentally. A special focus is put on revealing the contribution of various types of noise to the ambient noise associated with the gradient sensor. 

### 4.1. Evaluation of ME Voltage Coefficient Spectra

**Figure 2:**
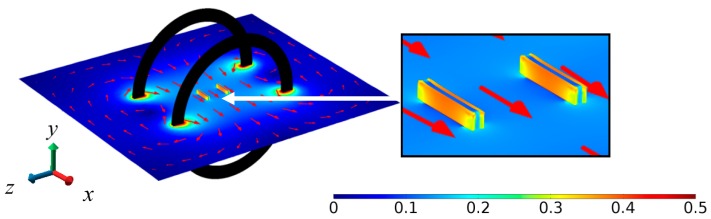
FEA result of magnetic field (|B|, mT) distribution in the central *x*–*z* plane involving the two ME composites separated by a baseline (*L*) of 35 mm in the axial (*z*-) direction and under the excitation of a pair of Helmholtz coils with |B| = 0.125 mT at *f* = 1 kHz in the transverse (*x*-) direction. The inset shows the zoomed-in view in the vicinity of the two ME composites. The red arrows indicate the directions of **B**.

**Figure 3:**
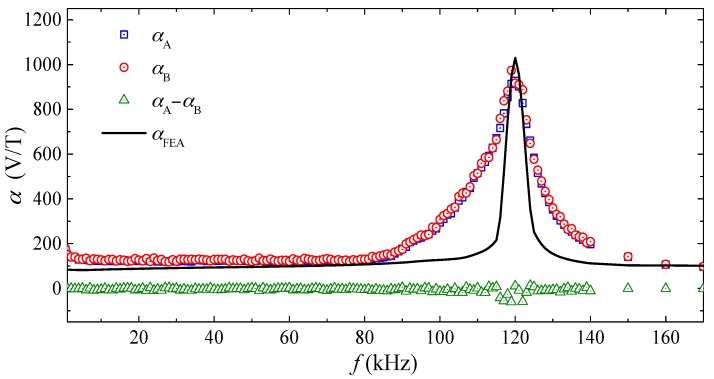
Frequency (*f*) dependence of measured ME voltage coefficients of the two ME composites ($\alpha_{A}$ and $\alpha_{B}$) and their difference ($\alpha_{A}-\alpha_{B}$), together with that of the ME voltage coefficient calculated from FEA ($\alpha_{\mathrm{FEA}}$).

Figure 2 shows the FEA result of magnetic field (|B|) distribution in the central *x–z* plane involving the two ME composites separated by a baseline (*L*) of 35 mm in the axial (*z*-) direction and under the excitation of a pair of Helmholtz coils with |B| = 0.125 mT at *f* = 1 kHz in the transverse (*x*-) direction in accordance with the experimental setup for the evaluation of the ME voltage coefficient spectra to be discussed in Figure 3. The zoomed-in view in the vicinity of the two ME composites indicates an in-phase deformation of the ME composites with a uniform B generated by the Helmholtz coils in the transverse (*x*-) direction. 

Figure 3 plots the frequency (*f*) dependence of the measured ME voltage coefficients of the two ME composites ($\alpha_{A\;}$ and $\alpha_{B\;}$) and their difference ($\alpha_{A}-\alpha_{B\;}$) for comparison with that of the ME voltage coefficient calculated from FEA ($\alpha_{\mathrm{FEA}}$). The $\alpha_{A}$ and $\alpha_{B\;}$ spectra were evaluated by producing an AC reference voltage of constant amplitude over the prescribed *f* range of 1 Hz–170 kHz using a lock-in amplifier (SRS SR865); by converting and amplifying the AC reference voltage into the corresponding AC current using a current supply amplifier (AE Techron 7548); by driving the Helmholtz coils with the AC current to generate **B** with a constant |B| = 0.125 mT in the transverse (*x*-) direction; and by measuring $V_{M,A}$ and $V_{M,B}$ using the lock-in amplifier. The AC current for generating **B** was monitored and assured using a current probe (HIOKI 9273) and a signal conditioner (HIOKI 3271) connected to the current feedback input of the lock-in amplifier. The $\alpha_{\mathrm{FEA}}$ spectrum was calculated by sweeping the solver frequency in COMSOL Multiphysics^®^ from 1 Hz to 170 kHz in steps of 100 Hz. It is seen from Figure 3 that the $\alpha_{A\;}$ and $\alpha_{B\;}$ spectra agree well with each other, and their difference ($\alpha_{A}\;-\alpha_{B}$) fluctuates very slightly (<3%) about zero in the frequency range of 1 Hz–170 kHz. This small difference confirms our design in Section 2 and Section 3, and supports the condition of $\alpha_{i,A}≅\alpha_{i,B}$ as stated in Equation (22) in the ME composites of our gradient sensor. Both $\alpha_{A\;}$ and $\alpha_{B\;}$ exhibit a large resonance peak of ~974 V/T at the resonance frequency ($f_{r}$) of 120 kHz, corresponding to a half longitudinal wavelength of 12 mm in the ME composites. Non-resonance $\alpha_{A\;}$ and $\alpha_{B\;}$ as large as 231 V/T are achieved from 1 Hz to 80 kHz. Comparing the measured $\alpha_{A\;}$ and $\alpha_{B\;}$ spectra with the calculated $\alpha_{\mathrm{FEA}}$ spectrum, the broader resonance width in both $\alpha_{A\;}$ and $\alpha_{B\;}$ can be explained by the presence of magneto-mechano-electrical losses in the ME composites, while the slight decrease in both $\alpha_{A}$ and $\alpha_{B}$ from 1 Hz to 80 kHz can be regarded as the existence of eddy-current loss in the conductive Terfenol-D plates. 

**Figure 4:**
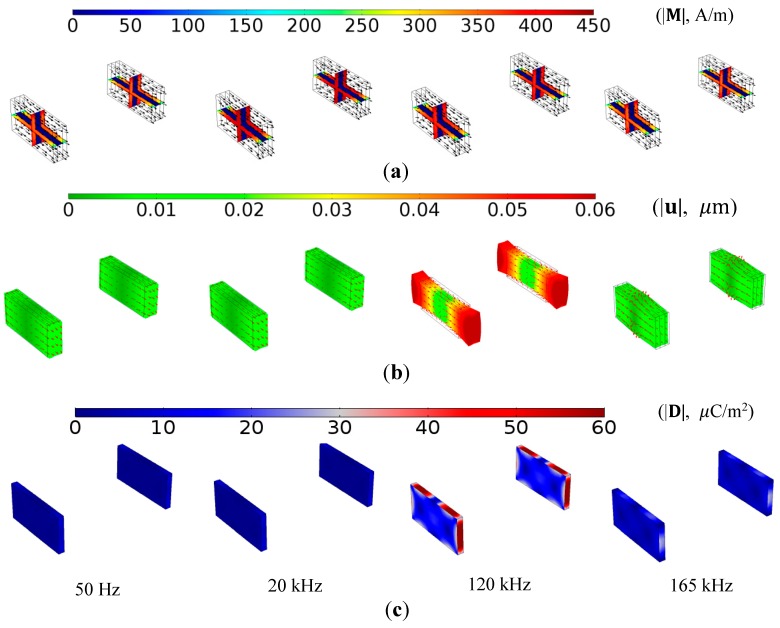
FEA results of ME composites under a uniform B at four different frequencies of 50 Hz, 20 kHz, 120 kHz, and 165 kHz: (**a**) AC magnetization (|M|, A/m) in rainbow colors and its directions in black arrows; (**b**) $5\times 10^{4}$-scaled total dynamic mechanical displacement ($\left|u_{\mathrm{MS}}\right|$ or $\left|u_{\mathrm{PE}}\right|$, μm) in traffic colors and its directions in red arrows; and (**c**) electric displacement field (|D|, μC/m^2^) in wave colors.

Figure 4 shows the FEA results of the AC magnetization (|M|), $5\times 10^{4}$-scaled total dynamic mechanical displacement ($\left|u_{\mathrm{MS}}\right|$ or $\left|u_{\mathrm{PE}}\right|$), and electric displacement field (|D|) of the two ME composites under a uniform B at four different *f* of 50 Hz, 20 kHz, 120 kHz, and 165 kHz. The |M| illustrations in Figure 4a reveal an even and high volume average of |M| in the Terfenol-D plates at lower frequency excitations of 50 Hz and 20 kHz. By contrast, a small volume average of |M| in the Terfenol-D plates and a large |M| on the surfaces of the Terfenol-D plates are seen at higher frequency excitations of 120 and 165 kHz because of the eddy-current-induced demagnetization. The observations are verified by further investigation into the volume average of |M| in the Terfenol-D plates, giving 386.16, 385.40, 363.20, and 349.58 A/m at the corresponding *f*. The decrease in volume average of |M| with increasing *f* provides an insight into the slight decrease in both $\alpha_{A}\;$ and $\alpha_{B}$ from 1 Hz to 80 kHz in Figure 3, and it can be ascribed to the weakening of the magnetomechanical coupling in the Terfenol-D plates as a result of the eddy-current-induced demagnetization as expressed in Equation (6). The $\left|u_{\mathrm{MS}}\right|$ or $\left|u_{\mathrm{PE}}\right|$ illustrations in Figure 4b indicate the domination of the in-phase longitudinal deformation in the two ME composites with a uniform B for *f* up to $f_{r}$ = 120 kHz. The deformation is maximized at 120 kHz and turns into out-of-phase with B for *f* above 120 kHz. The |D| illustrations in Figure 4c suggest that |D| in the two ME composites reaches the maximum value at 120 kHz. This agrees with the maximization of deformation at 120 kHz in Figure 4b because of the resonance ME effect [16]. 

### 4.2. Calibration of Detection Sensitivity

**Figure 5:**
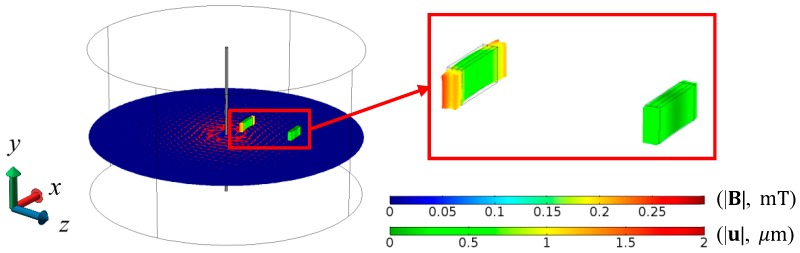
FEA results of magnetic field (|B|, mT) distribution and total dynamic mechanical displacement ($\left|u_{\mathrm{MS}}\right|$ or $\left|u_{\mathrm{PE}}\right|$, μm) of the two ME composites in accordance with the configuration and calibration of the gradient sensor in Figure 1a and in response to the excitation of a 2 m straight cable by an 1 A (RMS) AC current at 1 kHz. The inset shows the zoomed-in view of the two ME composites in $2\times 10^{6}$ scale.

Figure 5 shows the FEA results of the magnetic field (|B|) distribution and total dynamic mechanical displacement ($\left|u_{\mathrm{MS}}\right|$ or $\left|u_{\mathrm{PE}}\right|$) of the two ME composites in accordance with the configuration and calibration of the gradient sensor in Figure 1a. An AC current of 1 A RMS amplitude and 1 kHz frequency was injected into the straight cable of 2 m length to produce B. The two ME composites exhibit different levels of B-induced deformations along their lengths in the transverse (*x*-) direction due to the relative separation by the 35 mm baseline. This also reflects the case of using the gradient sensor in detecting transverse MFGs associated with current-carrying cables. The inset of Figure 5 reveals that the two ME composites are dominant by longitudinal modes with minor contribution of bending modes.

**Figure 6:**
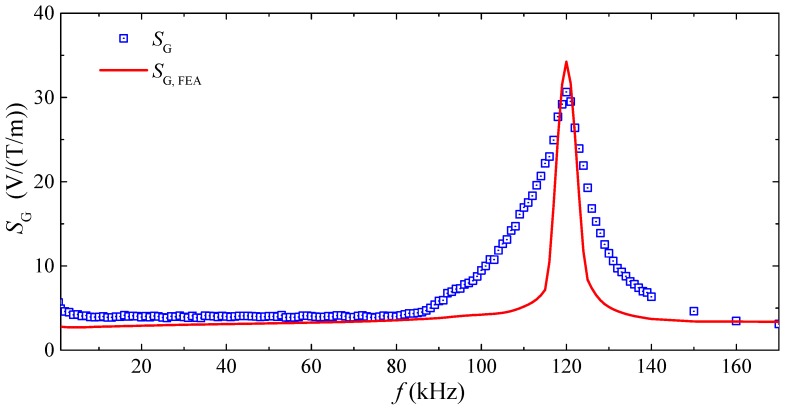
Frequency (*f*) dependence of measured and calculated detection sensitivities ($S_{G}$ and $S_{G,\;\mathrm{FEA}}$) of the gradient sensor.

Figure 6 plots the frequency (*f*) dependence of the measured and calculated detection sensitivities ($S_{G}$ and $S_{G,\;\mathrm{FEA}}$) of the gradient sensor. The $S_{G}$ spectrum was measured by fixing the gradient sensor in the *x–z* plane as in Figure 1 and Figure 5; by producing an AC reference voltage of constant amplitude over the prescribed *f* range of 1 Hz–170 kHz using a lock-in amplifier (SRS SR865); by converting and amplifying the AC reference voltage into the corresponding AC current using a current supply amplifier (AE Techron 7548); by driving the 2 m straight cable with the AC current of 0–12 A in steps of 0.5 A to generate a transverse MFG ($G_{xz}$) of 0–3.2 mT/m; and by measuring $V_{G}$ using the lock-in amplifier. The distance (*R*) from the center of the straight cable to the first ME composite was set at 15 mm, while the baseline (*L*) was 35 mm. The AC current for generating $G_{xz}$ was monitored and assured using a current probe (HIOKI 9273) and a signal conditioner (HIOKI 3271) connected to the current feedback input of the lock-in amplifier. The $S_{G,\;\mathrm{FEA}}$ spectrum was calculated by sweeping the solver frequency in COMSOL Multiphysics^®^ from 1 Hz to 170 kHz in steps of 100 Hz. The $S_{G}$ and $S_{G,\;\mathrm{FEA}}$ spectra are quantitatively similar to the $\alpha_{A\;}$ (or $\alpha_{B\;}$) and $\alpha_{\mathrm{FEA}}$ spectra in Figure 3, suggesting the determination of $S_{G}$ by $\alpha_{A\;}$ and $\alpha_{B\;}$, as prescribed by Equation (20). Large non-resonance $S_{G}$ of 7.53 V/T/m is achieved over a broad frequency range of 1 Hz–80 kHz, while high resonance $S_{G}$ of 30.6 V/(T/m) is obtained at $f_{r}$ of 120 kHz.

**Figure 7:**
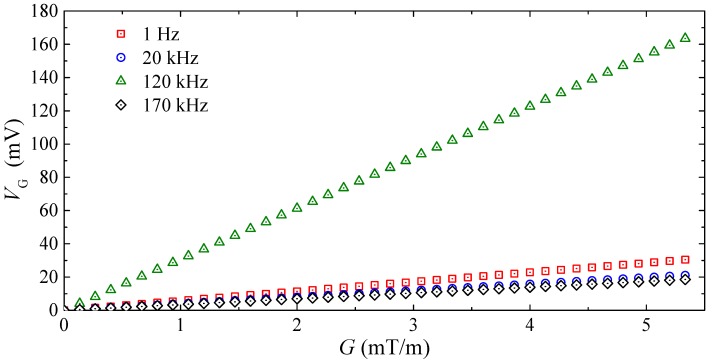
Measured gradient sensor output voltage ($V_{G}$) as a function of transverse MFG (G) at four different frequencies of 1 Hz, 20 kHz, 120 kHz, and 170 kHz.

Figure 7 gives the measured gradient sensor output voltage ($V_{G}$) as a function of transverse MFG (G) at four different frequencies of 1 Hz, 20 kHz, 120 kHz, and 170 kHz performed in the same measurement process of Figure 6. Among the four $V_{G}$–G curves, the one with the largest slope is obtained at 120 kHz, which is in good agreement with the measured $S_{G}$ spectrum in Figure 6. An analysis of the input-output nonlinearity for the G–$V_{G}$ responses over the *f* range of measurement finds a very small nonlinearity value of <10 ppm. This nonlinearity value is comparable to that of the fluxgate gradient sensors for space applications [32].

### 4.3. Evaluation of Voltage Noise Density Spectra and Gradient Noise Spectra

**Figure 8:**
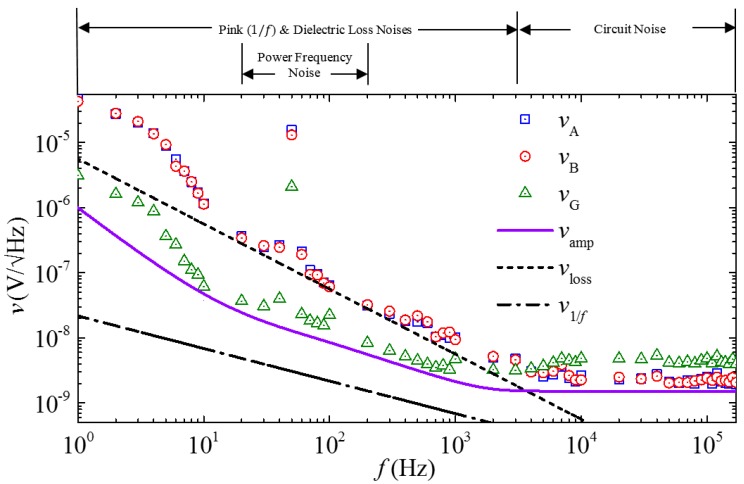
Measured voltage noise density spectra of the two ME composites ($v_{A}$ and $v_{B}$), the gradient sensor ($v_{G}$), and the lock-in amplifier noise floor ($v_{\mathrm{amp}}$), together with the calculated pink noise density ($v_{1/f}$) and dielectric loss noise density ($v_{\mathrm{loss}}$) spectra.

Figure 8 shows the measured voltage noise density spectra of the two ME composites ($v_{A}$ and $v_{B}$), the gradient sensor ($v_{G}$), and the lock-in amplifier noise floor ($v_{\mathrm{amp}}$), together with the calculated pink noise density ($v_{1/f}$) and dielectric loss noise density ($v_{\mathrm{loss}}$) spectra. The $v_{A}$, $v_{B}$, and $v_{G}$ spectra were independently measured from the output of the two ME composites and that of the gradient sensor, respectively, in a magnetically unshielded laboratory environment at 20 °C using a lock–in amplifier (SRS SR865) in the range of 1 Hz–170 kHz with a bandwidth (∆f) of 2.6 Hz [31]. The $v_{\mathrm{amp}}$ was measured by short-circuiting the input BNC terminal of the lock–in amplifier using the same instrument configuration described above. The $v_{1/f}$ and $v_{\mathrm{loss}}$ spectra were calculated using Equations (24) and (25) with the parameters listed in Table 3. Since the effect of the ambient electric field noise is minimized by the copper shield, the $v_{A}$, $v_{B}$, and $v_{G}$ spectra in Figure 8, in confirmation with the calculated $v_{1/f}$ and $v_{\mathrm{loss}}$ spectra, are dominated by the pink (1/f) noise and the dielectric loss noise from 1 Hz to 3 kHz. In the 20–150 Hz range, the power-frequency noise arising from the magnetically-unshielded laboratory environment has an obviously added effect on the background of the pink noise and the dielectric loss noise. In the further elevated frequency range of 3–170 kHz, the circuit noise associated with measurements is active since $v_{A}$, $v_{B}$, and $v_{G}$ all fluctuate over constant values. Nonetheless, the $v_{G}$ spectrum, in comparison with the $v_{A}$ and $v_{B}$, suggests that the MFG technique is a powerful technique to suppress various types of ambient noise, including pink noise, dielectric loss noise, and power-frequency noise, for f up to 3 kHz. Beyond 3 kHz, $v_{G}$ becomes double that of $v_{A}$ and $v_{B}$. This can be explained by the phase-lag between the voltage noises ($v_{A}\sqrt{∆f}$ and $v_{B}\sqrt{∆f}$) in the two ME composites at high *f* because the voltage noises have random phases at high *f* (i.e., f > 3 kHz).

**Figure 9:**
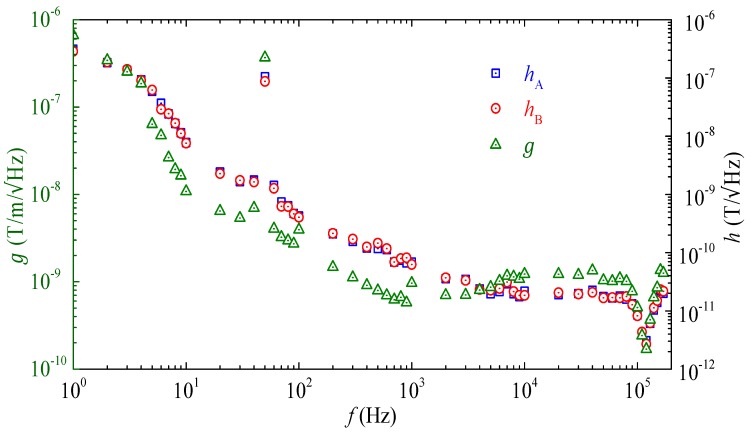
Field noise ($h_{A}$ and $h_{B}$) spectra of the two ME composites and gradient noise (g) spectrum of the gradient sensor.

Figure 9 shows the field noise ($h_{A}$ and $h_{B}$) spectra of the two ME composites and the gradient noise (g) spectrum of the gradient sensor. The $h_{A}$, $h_{B}$, and g spectra were evaluated using the measured $v_{A}$, $v_{B}$, and $v_{G}$ spectra in Figure 8 as well as Equations (26) and (27). It is found that the ME composites have $h_{A}$ and $h_{B}$ of 0.003–320 nT/$\sqrt{\mathrm{Hz}}$ and the gradient sensor has g of 0.16–620 nT/m/$\sqrt{\mathrm{Hz}}$, both in the frequency range of 1 Hz–170 kHz in a magnetically-unshielded laboratory environment at 20 °C. The results suggest that the gradient sensor can be used for transverse magnetic field detection with a field noise of 0.003–320 nT/$\sqrt{\mathrm{Hz}}$ as well as for transverse MFG detection with a gradient noise of 0.16–620 nT/m/$\sqrt{\mathrm{Hz}}$. The performance of $h_{A}$, $h_{B}$, and g can be further increased by using ME composites with a higher ME voltage coefficient ($\alpha_{A\;}$ and $\alpha_{B\;}$).

### 4.4. Evaluation of Spatial Transfer Function

**Figure 10:**
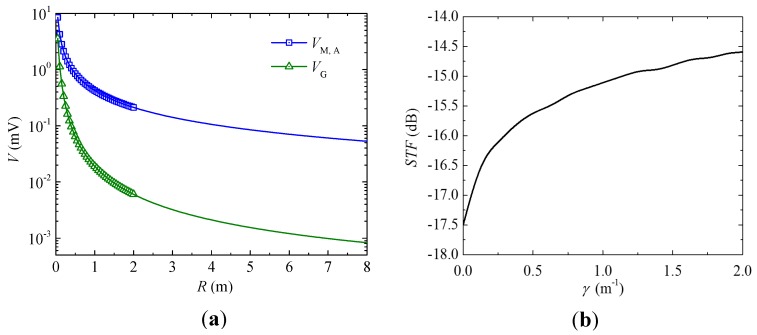
(**a**) Measured (symbol) and fitted (line) output voltages of the first ME composite ($V_{M,A}$) and the gradient sensor ($V_{G}$) at various distances (*R*) from the center of the straight cable to the first ME composite. (**b**) Spatial transfer function (*STF*) of the gradient sensor on a decibel scale.

Figure 10a shows the measured $V_{M,A}$ and $V_{G}$ as a function of *R* in response to the artificially generated common-mode magnetic field noise by the straight cable as in Figure 1a. Figure 10b plots the spatial transfer function (*STF*) of the gradient sensor as a function of spatial frequency (γ). The $V_{M,A}$ and $V_{G}$ in Figure 10a were measured by holding a straight cable with a length of 8 m along the *y*-direction as in Figure 1a, by passing an AC current of 12 A RMS amplitude and 50 Hz frequency through the straight cable, and by fixing the gradient sensor in the *x–z* plane to enable its movement along the axial (*z*-) direction with adjustable distance (*R*) from the center of the straight cable to the first ME composite. The measured output voltage of the first ME composite ($V_{M,A}$) and that of the gradient senor ($V_{G}$) were independently measured using the lock-in amplifier at various *R* of 0.05–2 m in steps of 0.05 m. The *STF* in Figure 10b was then obtained by dividing the Fourier transform of the spline-interpolated $V_{G}–R$ curve over that of the spline-interpolated $V_{M,A}–R$ curve, analogous to the traditional method used for evaluating the *STF* of existing axial gradient sensors [33,34]. The *STF* in Figure 10b indicates that our gradient sensor is essentially a high-pass spatial filter with a minimal *STF* value of –17.5 dB when γ=0 (i.e., *R* →∞) and an increased *STF* value of <–14.5 dB when γ is within the range of 0–2 m^−1^ (i.e., *R* ≥ 0.5 m). This means that our gradient sensor has a common-mode magnetic field noise rejection rate better than –14.5 dB (for *R* ≥ 0.5 m) under the interference of remote common-mode magnetic field noise sources.

**Figure 11:**
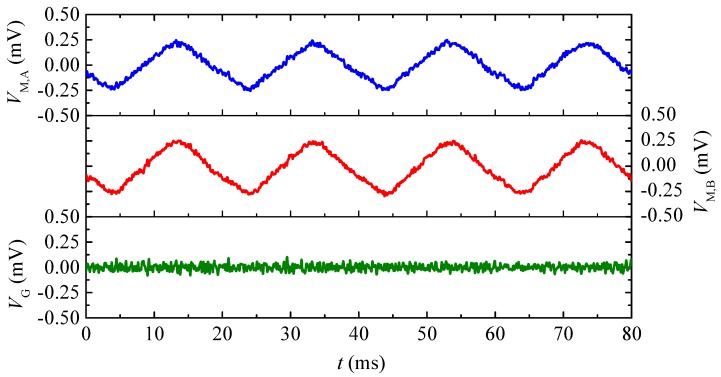
ME voltage waveforms of the two ME composites ($V_{M,A}$ and $V_{M,B}$) and output voltage waveform of the gradient sensor ($V_{G}$) in response to a common-mode ambient power-frequency noise created by passing an AC current of 12 A RMS amplitude and 50 Hz frequency through a 2 m straight cable having a 1.8 m separation from the gradient sensor.

Figure 11 shows the ME voltage waveforms of the two ME composites ($V_{M,A}$ and $V_{M,B}$) and the output voltage waveform of the gradient sensor ($V_{G}$) when *R* = 1.8 m. The waveforms of $V_{M,A}$, $V_{M,B}$, and $V_{G}$ were recorded using a mixed signal oscilloscope (Tektronix MSO2014) with a 60 kHz low-pass filter. This is to demonstrate the common-mode ambient noise suppression performance of the gradient sensor. It is seen that the common-mode voltage noises, which are induced by the common-mode magnetic field noise and picked up by the two ME composites, are suppressed by 36 times in the gradient sensor. 

## 5. Conclusions

We have developed a small-scale and standalone ME transverse gradient sensor for a passive and direct detection of MFGs transverse to its length into electrical voltages while simultaneously suppressing common-mode ambient noise by combining the key advantages of the ME effect and the MFG technique in a pair of magnetically-biased, electrically-shielded, and mechanically-enclosed Terfenol-D/PZT/Terfenol-D trilayer ME composites having a transverse orientation and an axial baseline. The design and modeling processes of the gradient sensor have been established. A characteristic design of the gradient sensor has been reported, both theoretically and experimentally, to simultaneously demonstrate a high detection sensitivity of 0.4–30.6 V/(T/m), a strong common-mode magnetic field noise rejection rate of <–14.5 dB, a small input-output nonlinearity of <10 ppm, and a low gradient noise of 0.16–620 nT/m/$\sqrt{\mathrm{Hz}}$ over a broad frequency range of 1 Hz–170 kHz under a small baseline of 35 mm in a magnetically-unshielded laboratory environment. The gradient sensor is especially useful to suppress pink (1/*f*) noise, dielectric loss noise, and power-frequency noise below 3 kHz. The high detection performance, in conjunction with the passive, direct, and broadband MFG detection ability in a small-scale and standalone package, makes the gradient sensor a promising transverse MFG detection device in contrast to traditional fluxgate, Hall, and SQUID gradient sensors.

## Figures and Tables

**Table 1:** Terfenol-D material parameters.

Parameter	Symbol (SI Unit)	Value					
Electrical Conductivity	σ (S/m)	1.67 × 10^6^					
Density	ρ (kg/m^3^)	9200					
Saturation Magnetization	$M_{s}$ (kA/m)	132					
Saturation Magnetostriction	$\lambda_{001}$ (ppm) $\lambda_{111}$ (ppm)	1200 800					
Magnetic Susceptibility	$\chi_{m}$	7.1					
4th Order Elastic Matrix	$C^{H}$ (GPa)	70.2	20.4	23.4	0	0	0
		20.4	70.2	23.4	0	0	0
		23.4	23.4	81.5	0	0	0
		0	0	0	6.4	0	0
		0	0	0	0	6.4	0
		0	0	0	0	0	6.4

**Table 2:** PZT material parameters.

Parameter	Symbol (SI Unit)	Value					
Electrical Conductivity	σ (S/m)	$10^{-20}$					
Density	ρ (kg/m^3^)	7600					
Permittivity	dia $\epsilon_{r}$	904, 904, 561					
Stress–Charge Piezoelectric Coupling Matrix	$e^{E}$ (C/m^2^)	0	0	0	0	17	0
		0	0	0	17	0	0
		−6.6	−6.6	23	0	0	0
4^th^ Order Elastic Matrix	$C^{E}$ (GPa)	132	72.9	72.9	0	0	0
		72.9	132	72.9	0	0	0
		72.9	72.9	118.5	0	0	0
		0	0	0	28.2	0	0
		0	0	0	0	28.2	0
		0	0	0	0	0	29.6

**Table 3:** Parameters for calculating pink (1/f) noise density and dielectric loss noise density spectra.

Property, (SI Unit)	Value
Temperature, T (K)	293.15
Band-width, ∆f (Hz)	2.6
Resistance of load circuit, $R_{L}$ (MΩ)	100
Capacitance of ME composite, *C* (pF)	580
Dielectric loss factor, tanδ (%)	0.15
